# A New Equation to Estimate Glomerular Filtration Rate in Chinese Elderly Population

**DOI:** 10.1371/journal.pone.0079675

**Published:** 2013-11-11

**Authors:** Xun Liu, Yanni Wang, Cheng Wang, Chenggang Shi, Cailian Cheng, Jinxia Chen, Huijuan Ma, Linsheng Lv, Lin Li, Tanqi Lou

**Affiliations:** 1 Division of Nephrology, Department of Internal Medicine, The Third Affiliated Hospital of Sun Yat-sen University, Guangzhou, China; 2 College of Biomedical Engineering, South China University of Technology, Guangzhou, China; 3 Operating Room, The Third Affiliated Hospital of Sun Yat-sen University, Guangzhou, China; 4 Department of Laboratory Medicine, The Third Affiliated Hospital of Sun Yat-sen University, Guangzhou, China; University of Washington, United States of America

## Abstract

**Background:**

We sought to develop a new equation to estimate glomerular filtration rate (GFR) in Chinese elderly population.

**Methods:**

A total of 668 Chinese elderly participants, including the development cohort (n = 433), the validation cohort (n = 235) were enrolled. The new equation using the generalized additive model, and age, gender, serum creatinine as predictor variables was developed and the performances was compared with the CKD-EPI equation.

**Results:**

In the validation data set, both bias and precision were improved with the new equation, as compared with the CKD-EPI equation (median difference, −1.5 ml/min/1.73 m^2^ vs. 7.4 ml/min/1.73 m^2^ for the new equation and the CKD-EPI equation, [P<0.001]; interquartile range [IQR] for the difference, 16.2 ml/min/1.73 m^2^ vs. 19.0 ml/min/1.73 m^2^ [P<0.001]), as were accuracies (15% accuracy, 40.4% vs. 30.6% [P = 0.02]; 30% accuracy, 71.1% vs. 47.2%, [P<0.001]; 50% accuracy, 90.2% vs. 75.7%, [P<0.001]), allowing improvement in GFR categorization (GFR category misclassification rate, 37.4% vs. 53.2% [P = <0.001]).

**Conclusions:**

A new equation was developed in Chinese elderly population. In the validation data set, the new equation performed better than the original CKD-EPI equation. The new equation needs further external validations. Calibration of the GFR referent standard to a more accurate one should be an useful way to improve the performance of GFR estimating equations.

## Introduction

The world population is rapidly ageing. Between 2000 and 2050, the proportion of the world's population aged 60 years and over will double from about 11% to 22%. The absolute number of people aged over 60 years is expected to increase from 605 million to 2 billion over the same period [1]. Populations in China are rapidly aging, too. Elderly population in China will increase from 178 million in 2011 to 221 million in 2015, with an average increase of 8.6 million older people annually [2]. Glomerular filtration rate (GFR) decreases with age [3], resulting from both physiologic aging of the kidney and specific pathologic influences. Accurate determination of GFR is important for the diagnosis and categorization of chronic kidney disease (CKD), as well as adjustment of therapeutic interventions [4]. By far, serum creatinine (SC) remains the most commonly used endogenous filtration marker of kidney function. However, most of the SC based GFR prediction equations were not initially developed in the elderly. In a previous study, we found that the Cockroft-Gault equation, the Modification of Diet in Renal Disease (MDRD) equations, the Chronic Kidney Disease Epidemiology Collaboration (CKD-EPI) equation, the Jelliffe-1973 equation, and the Hull equation were not suitable for estimating GFR in the elderly Chinese population investigated [5]. To date, no equation has been recommended to evaluate GFR in the elderly population. In this study, we developed a new equation in Chinese elderly population and compare its performances with the CKD-EPI equation in GFR estimation.

## Methods

### Participants

Participants who were older than 60 years were selected to measure GFR at the Third Affiliated Hospital of Sun Yat-sen University, Guangzhou, China from Jan 2005 to Dec 2011. Patients with acute kidney function deterioration, clinical edema, skeletal muscle atrophy, pleural effusion or ascites, malnutrition, amputation, heart failure or ketoacidosis were excluded from the study. Participants that were taking cimetidine or trimethoprim were excluded as well. No subject was being treated with dialysis at the time of the study. Exclusion criteria were described elsewhere [6]. CKD was diagnosed and categorized according to the National Kidney Foundation Disease Outcomes Quality Initiative (NKF-K/DOQI) clinical practice guidelines [7]. The institutional review board of the Third Affiliated Hospital of Sun Yat-sen University approved the study. Written informed consent was obtained from each participant. A total of 668 elderly participants were included in this study, including 398 men and 270 women. The mean age was 70.0±6.7 yr (ranged from 60 to 93 yr), and the mean measured GFR (mGFR) was 51.2±26.0 ml/min/1.73 m^2^ (ranged from 6.6 to 134.1 ml/min/1.73 m^2^). Causes and GFR categories are listed in Table 1. The total cohort was randomly divided into the development data set (n = 433) and the validation data set (n = 235).

**Table 1:** Participant characteristic.

Characteristic (N = 668)	Mean (standard deviation) or number (percentage)
Age (year)	70.1(6.7)
Male sex [n (%)]	398(59.6)
Body mass index (kg/m^2^)	23.6(3.7)
Weight (kg)	61.9(11.7)
Height (cm)	161.7(8.2)
Body-surface area (m^2^)	1.7(0.2)
Serum creatinine (mg/dl)	2.4(2.0)
Causes [n (%)]	
Diabetic nephropathy	280(41.9)
Hypertension	114(17.1)
Chronic tubulointerstitial disease	45(6.7)
Primary glomerular disease	68(10.2)
Renovascular disease	42(6.3)
polycystic kidney disease	11(1.6)
Other causes or causes unknown	78(11.7)
Healthy volunteers	30(4.5)
Measured GFR	
Mean (ml/min/1.73 m^2^)	51.2(26.0)
<15 (ml/min/1.73 m^2^)	60(9.0)
15–29 (ml/min/1.73 m^2^)	174(26.0)
30–59 (ml/min/1.73 m^2^)	266(39.8)
60–89 (ml/min/1.73 m^2^)	139(20.8)
>90 (ml/min/1.73 m^2^)	29(4.3)

Abbreviations: GFR, glomerular filtration rate.

### Laboratory measurements

GFR was measured by the ^99m^Tc-DTPA renal dynamic imaging method [8]–[9], as described previously [10]. In order to ensure that our mGFR values were calibrated equally to the dual plasma sample ^99m^Tc-DTPA [11] GFR, the minimum sample size based on the results in a previous study [12] was calculated as 36 (95% confidence level and 80% power using Open Epi Version 2 [13]). After image acquisition, heparin anti-coagulated blood samples were taken 2 and 4 h after injection from the opposite forearm. Plasma was separated, and radioactivity in the plasma was counted in multi-function well counter (ZD-6000 multi-function instrument from Zhida Technology Company, Xian, China). SC was determined by the enzymatic method on a Hitachi 7180 autoanalyzer (Hitachi, Tokyo, Japan; reagents from Roche Diagnostics, Mannheim, Germany), and traceable to the isotope dilution mass spectrometry. We compared the new equation with the CKD-EPI equation [14].

### Statistical analysis

The difference between estimated GFR (eGFR) and mGFR was defined as eGFR minus mGFR. Precision was defined as the interquartile range (IQR) for the difference. Accuracy was measured as the percentage of eGFR not deviating more than 15%, 30% and 50% from the mGFR. The GFR category misclassification rate was determined as the proportion of patients misclassified into the correct NKF-K/DOQI GFR category. A Wilcoxon Mann-Whitney test, bootstrap method and McNemar test were used to compare the difference, IQR for difference and accuracy, respectively. All statistics were performed using SPSS software (version 11.0 SPSS, Chicago IL, USA) and Matlab software (version 2011b The Mathworks, Boston MA, USA).

## Results

### Calibration of the ^99m^Tc-DTPA renal dynamic imaging GFR

We randomly selected 36 participants (mean age 69.5±7.8 yr, ranged from 60 to 93 yr) in this study [(mGFR (ranged from 7.3 to 80.0 ml/min/1.73 m^2^) and performed dual plasma samples method 99mTc-DTPA clearance simultaneously with the renal dynamic imaging. In this cohort, the dynamic method underestimated referent GFR by 6.75 ml/min/1.731m^2^. The ^99m^Tc-DTPA renal dynamic imaging GFR value that was measured in our study can be calibrated to the dual plasma samples ^99m^Tc-DTPA clearance GFR using a linear regression equation: dual plasma sample ^99m^Tc-DTPA-GFR (ml/min/1.73 m^2^) = 1.503+1.137* ^99m^Tc-DTPA renal dynamic imaging-GFR (ml/min/1.73 m^2^) (R^2^ = 0.799, P<0.001).

### Development of the new equation

The new equation was developed from the development data set of this study by the generalized additive model method. The equation used age, gender and SC as the three predictor variables. The selection of knot and forms of smooth functions was the same as for the CKD-EPI equation. The coefficients were estimated by least-square error (Table 2).

**Table 2:** CKD-EPI equation and the new equation.

Basis of equation and sex	Serum creatinine	Equation for estimating GFR
CKD-EPI equation		
Female	≤0.7 mg/dl	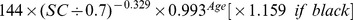
Female	>0.7 mg/dl	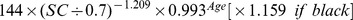
Male	≤0.9 mg/dl	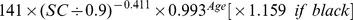
Male	>0.9 mg/dl	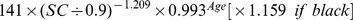
New equation		
Female	≤0.7 mg/dl	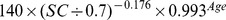
Female	>0.7 mg/dl	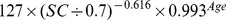
Male	≤0.9 mg/dl	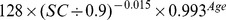
Male	>0.9 mg/dl	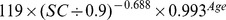

### Performance of the equations

In the validation data set, both bias and precision were improved with the new equation, as compared with the CKD-EPI equation (median difference, −1.5 ml/min/1.73 m^2^ vs. 7.4 ml/min/1.73 m^2^ for the new equation and the CKD-EPI equation, [P<0.001]; interquartile range [IQR] for the difference, 16.2 ml/min/1.73 m^2^ vs. 19.0 ml/min/1.73 m^2^ [P<0.001]), as were accuracies (15% accuracy, 40.4% vs. 30.6% [P = 0.02]; 30% accuracy, 71.1% vs. 47.2%, [P<0.001]; 50% accuracy, 90.2% vs. 75.7%, [P<0.001]), allowing improvement in GFR categorization (GFR category misclassification rate, 37.4% vs. 53.2% [P = <0.001]) (Table 3).

**Table 3:** Performance of bias, precision and accuracy between measured GFR and estimated GFR in the validation data set.

Variable	Measured GFR (ml/min/1.73 m^2^)			
	Overall	<30	30–59	≥60
Bias – median difference (ml/min/1.73 m^2^)				
CKD-EPI equation	7.4	8.8	3.9	8.0
New equation	−1.5	−4.4	−3.7	10.2
Precision – IQR of the difference (ml/min/1.73 m^2^)				
CKD-EPI equation	19.0	9.3	23.2	31.7
New equation	16.2	8.7	16.4	25.0
Accuracy - %				
15% accuracy				
CKD-EPI equation	30.6	12.7	36.2	38.8
New equation	40.4	38.1	40.0	43.3
30% accuracy				
CKD-EPI equation	47.2	23.8	48.6	67.2
New equation	71.1	61.9	70.5	80.6
50% accuracy				
CKD-EPI equation	75.7	58.7	75.2	92.5
New equation	90.2	73.0	95.2	98.5
GFR category misclassification - %				
CKD-EPI equation	53.2	60.3	47.6	55.2
New equation	37.4	41.3	26.7	50.7

Abbreviations: GFR, glomerular filtration rate; CKD-EPI, Chronic Kidney Disease Epidemiology Collaboration; IQR, interquartile range.

## Discussion

The CKD-EPI equation is favored in the North America, Europe, and Australia and is recommended as a comparator for new equations in all locations [15]. However, studies found that the CKD-EPI equation was less accurate in Chinese elderly populations [5], [16]. In this study, we developed a new equation for Chinese elderly population GFR estimation by using the same generalized additive model, input variables and methods of SC measurement as those in the CKD-EPI equation. While comparing with the CKD-EPI equation, this new equation exhibited significant improvement of either bias or precision in the validation data set, which indicating this new equation might allow for greater accuracy in GFR categorization. Such results can help the clinicians to make proper diagnostic and therapeutic interventions while treating patients from the Chinese elderly population.

The ^99m^Tc-DTPA renal dynamic imaging is simple, fast and less expensive for the determination of GFR, and is widely used as a reference method for GFR estimation in China. However, some studies have proven that the renal dynamic imaging may result in great bias [11]–[12]. The dual plasma sample method is recognized as a more accurate method for the measurement of GFR and was recommended by the Nephro-urology Conference [17]. Calibration of both serum creatinine and cyctatin C assays have been successfully applied in the field of GFR estimation [14], [18]. In this study, for the first time we calibrated our mGFR to the dual plasma sample ^99m^Tc-DTPA-GFR. While the difference between the dual method and the dynamic method we found was not similar in direction and amount to that reported by Peng Xie et al [11]. This highlignts the limitation of the variability among clinical laboratories in measurement of GFR. We recommend the use of a standardized GFR measurement such as inulin renal clearance for calibration.

Why is the new equation superior to the original CKD-EPI one? First, characteristics of participants in the development population as well as the validation population of two equations were quite different. The mean age in our study was 70.1 years, which was much older than that in the CKD-EPI equation. And the mean mGFR in our study was much lower than that in the CKD-EPI equation, too. Differences in the study population lead to errors in the comparison between two GFR estimating equations [19]. Second, the method to measure GFR was different. Urinary clearance of iothalamate was used for the development and internal validation of the CKD-EPI equation. However, DTPA renal dynamic imaging method used in this study was calibrated to the dual plasma sample DTPA method. Variability in the method to measure GFR introduces bias in the estimation of GFR [20].

There are several limitations in this study. First, the new equation needs further external validations to evaluate its applications. Second, the mean mGFR in our study was relatively low, thus might not favor participants with early kidney damage. Third, the percentage of healthy volunteers was relatively small in our study. The new equation's performance in the elderly with normal or near normal kidney function needs further evaluation.

In conclusion, a new equation was developed in Chinese elderly population. In the validation data set, the new equation performed better than the original CKD-EPI equation. The new equation needs further external validations. Calibration of the GFR referent standard to a more accurate one should be a useful way to improve the performance of GFR estimating equations.
